# Representation of interactional metadiscourse in translated and native English: A corpus-assisted study

**DOI:** 10.1371/journal.pone.0284849

**Published:** 2023-07-27

**Authors:** Isabelle Chou, Weiyi Li, Kanglong Liu

**Affiliations:** 1 School of Foreign Languages, University of Electronic Science and Technology of China, Chengdu, China; 2 Department of English, The Chinese University of Hong Kong, Hong Kong, China; 3 Department of Chinese and Bilingual Studies, The Hong Kong Polytechnic University, Hong Kong, China; Chulalongkorn University, THAILAND

## Abstract

The present study aimed to investigate the differences between translated and non-translated English texts with regard to interactional metadiscourse features, which are crucial in engaging readers in the reasoning process and establishing the credibility of a proposition. Despite numerous studies investigating lexical and syntactic differences between translated and non-translated language, little research has been conducted on the textual level in terms of metadiscourse use. To address this gap, we conducted a comparative analysis of six interactional markers across two comparable multi-genre corpora, namely, FLOB (Freiburg-LOB Corpus of British English) comprising native English and the English subset of COCE (Corpus of Chinese-English) containing translated English. Our ANOVA analyses revealed that translated English exhibited a tendency to underuse stance features, such as hedges, boosters, and attitude markers, compared to native English. Furthermore, our post-hoc analysis revealed that genre modulated the use of metadiscourse features in both translated and native texts. Importantly, we found that there was greater cross-genre variation in the use of interactional metadiscourse in translated English than in native English. Our study highlights the unique characteristics of translation and emphasizes the importance of taking into account metadiscourse in the field of translation studies.

## 1. Introduction

Translated language has distinct characteristics compared to non-translated or spontaneous language, which is often referred to as "the third code" [1] or "translationese." The term "translationese" often carries a negative connotation, referring to linguistic elements that result from mechanical or word-for-word translation [2], or even inaccurate or incompetent translation [3]. "The third code," on the other hand, is used in a more neutral context, referring to subtle deviations from target language norms that are worthy of systematic investigation [1]. It is seen as a code in its own right, with its own standards and structural characteristics, though they are necessarily influenced by the source language and target language parameters [4]. Translation Studies has long sought to understand whether translations are systematically different from originally produced texts. By comparing translated and non-translated texts, researchers have identified some unique features of translations in order to gain a better understanding of translation as a distinct form of communication [5,6]. This has led to the concept of "translation universals," or TUs, which refer to features that are present in translated texts and are not the result of interference from specific linguistic systems [3]. The most commonly studied TUs are simplification (the tendency for translations to simplify language use compared to native texts), normalization (the tendency to conform to linguistic characteristics typical of the target language), explicitation (the tendency to state information more explicitly than in native texts), and conservatism (the tendency to conform to linguistic features typical of the target language) [6].

Over the years, researchers in this line of inquiry have studied how translations differ from non-translations by comparing various linguistic forms, such as lexical density, lexical variety, and information capacity [7–10]. However, relatively little research has been conducted on the use of metadiscourse in translated versus non-translated languages. Metadiscourse, which refers to language that helps convey the intended meaning and tone of the original text and signals the speaker’s or writer’s perspective on the topic being discussed [11,12], can be particularly important in translation as it helps ensure that the translated text is coherent, intelligible, and persuasive to readers [13,14]. However, translating metadiscourse can be challenging when working with languages that have different cultural norms and conventions for expressing it. Some studies have found that metadiscourse is less frequently used in translated languages [15–17], suggesting that translated languages may be less aware of the processing needs of the audience, while other studies have found no statistical difference in the distribution of metadiscourse between translated and non-translated languages [18,19]. Given the significance of metadiscourse in conveying meaning and perspective in communication, it is important to investigate how it is used and distributed in translated languages in comparison to source languages and non-translated languages. By studying the usage of metadiscourse in translated texts, we can gain insight into how well the translated text is able to fulfill the interpersonal and textual metafunction [20], which involves using language to engage the audience and convey the speaker’s or writer’s stance or perspective on a topic. Understanding the differences and similarities in the use of metadiscourse between translated and non-translated languages can also help improve the effectiveness of translation and facilitate successful communication between speakers of different languages.

In the quest of identifying translation universals, also known as the third code, several factors such as genre and text type may impact the frequency and usage of linguistic or metadiscourse features in translated language [21,22]. However, prior studies have mostly focused on specific genres such as literary [23] or legal texts [24], which limits the scope of their findings and raises questions about their consistency and generalizability. Therefore, to gain a better understanding of the patterns and potential variables at play, it is crucial to use a corpus of translated and non-translated language that is balanced across genres. Moreover, while some scholars have suggested disregarding the source language in the exploration of translation universals, recent studies have still placed emphasis on the source-target language pair [25,26]. Despite the significance of metadiscourse in conveying interpersonal meanings for accurate translations, no academic research has investigated the use of metadiscourse features in translations between English and Chinese, which are typologically distant languages. Hence, this research gap is particularly noteworthy.

It is crucial to examine whether there are any noticeable patterns in the use of metadiscourse features in translated English from Chinese that differ from English used spontaneously across various genres, including fiction, non-fiction, academic prose, and news. By analyzing a large corpus of translation data that includes a range of these genres, corpus-based studies can provide deeper insight into how metadiscourse features contribute to the realization of the interpersonal metafunction in translated English from Chinese and how these patterns may differ from those in non-translated English. This type of study can also enhance our understanding of the genre sensitivity of translated language features. With this broad context in mind, the current study employed a corpus-based approach to compare the usage and distribution of metadiscourse features in translated English from Chinese (using the Corpus of Chinese-English or COCE) with original English (using the Freiburg-LOB Corpus of British English or FLOB).

## 2. Metadiscourse features of translated languages

Metadiscourse is a term used to describe the way language refers to itself and includes linguistic resources that help readers interpret, evaluate, and organize what is being said. These features, which are deeply rooted in the scholarship of Jacobson’s "metalinguistic function" [27] and Halliday’s "metaphenomena" [28], play a crucial role in allowing speakers and writers to interact with their audience in a way that follows social and cultural norms [29,30]. From a metafunction perspective, metadiscourse represents the interpersonal and textual meanings of a message or text that need to be conveyed in a credible and convincing manner to be accepted and recognized by the audience of a particular community [30,31]. As posited by Hyland [32], writers employ interactive markers to explicitly convey their preferred information. These markers encompass transitions, frame markers, endophoric markers, evidentials, and code glosses. Conversely, interactional resources highlight writer-reader interactions and facilitate the transmission of the writers’ intentions to their readers. Six archetypal interactional markers are identified, namely hedges, boosters, attitude markers, engagement markers, self-mentions, and reader pronouns. Previous studies revealed that the use of metadiscourse features varies across genres and languages [33–35]. In addition, the field of translation has also begun to utilize metadiscourse frameworks to examine possible patterns in translated languages. As a bridge and mediator between two different languages, translators play a dual role in the production of translated texts: they are both the reader of the source text and the author of the translated text. During the translation process, it is very likely that the propositional content of the source text can often be reproduced in the translation, while the interactional content may be differently characterized due to different sociocultural norms and practices.

Metadiscourse, which includes both textual and interpersonal markers, is often found to be underrepresented in translated texts compared to non-translated texts. This has been observed in a number of studies that used different approaches to analyzing metadiscourse. For example, Herriman [16] studied the frequency of metadiscourse in non-translated Swedish non-fictional texts compared to Swedish texts translated from English, and found that there was a higher frequency of both textual and interactional markers in the non-translated texts. Kuhi [36] also found that translated Persian used significantly fewer metadiscourse items when compared to American presidential debate transcripts, using an interpersonal model of metadiscourse. Research efforts focusing on particular metadiscourse markers have consistently reported a decline in the frequency of their use in translated texts, thereby highlighting a noteworthy association between the act of translation and the usage of such linguistic features in discourse. Peterlin [17] compared the use of three textual markers ("article," "paper," and "here") in academic discourse translated from Slovene into English and comparable original English texts, and found that these markers were used far more frequently in the original texts. Mardani [37] found that Iranian writers used significantly more interpersonal markers than Iranian translators in persuasive texts like newspaper articles. Peterlin [38] also observed a lower frequency of engagement markers in English translations of Slovene academic texts compared to non-translated Slovene texts. Translation trainees have also been found to omit or modify hedges more frequently in their translations of English newspaper commentaries [39] and to use fewer stance markers in their translations compared to non-translated texts [40]. In addition, modal markers with a hedging reference are underrepresented in medical abstracts translated from English into French [41]. However, not all studies have found a significant difference in the use of metadiscourse markers between translated and non-translated texts. Farahani and Kazemian [18] found no significant difference in the distribution of interactive and interactional metadiscourse features in spoken Persian political discourse translated from English and original spoken Persian language. Overall, the research suggests that metadiscourse is often underrepresented in translated texts compared to non-translated texts, although the specific metadiscourse markers that are most affected and the extent of the underrepresentation can vary. It is pertinent to note that some studies have employed a limited sample size, which may compromise the reliability and generalizability of their findings. In addition, certain studies lacked transparency with regard to the total word count, which may lead to potential inaccuracies. A significant portion of the literature focuses exclusively on metadiscourse features in specific genres of non-literary texts, such as news reports [37,39] and academic texts [38,42,43], with only a few studies undertaking comparative analyses across different genres. Chung’s [35] research has revealed notable variations in the use of interactive and interactional metadiscourse in letters and reports. Consequently, the current body of literature presents some contradictory findings concerning the representation of metadiscourse features across various genres in translated texts. For example, Peterlin [17] found that not all textual metadiscourse items in original texts were translated into English, and that the English translations contained metadiscourse items not found in the original texts. In addition, non-translated English texts were found to have more metadiscourse markers than English texts translated from Slovene. Other research has also found that rhetorical markers were frequently omitted in Slovene translations of research papers by trainee translators [44]. Studies have shown that metadiscourse is often underrepresented in translated news texts compared to the original non-translated versions. For example, Mardani [37] found that there were more instances of metadiscourse in non-translated Iranian news articles compared to those translated from English. Other research has also found that there are often omissions and modifications of hedging devices, which are words or phrases that express hesitation or uncertainty, in journalistic texts translated from English into Slovene [39].

In the genre of general prose, Skrandies [45] discovered that writer-reader interaction, which refers to the way an author communicates with their audience through language, is characterized differently in German and English translations. Specifically, she found that translators tended to reproduce forms of metadiscourse in their English translations that differed in terms of the author’s presence and the level of reader involvement compared to the source text. Herriman [16] also found that translations and native writing have different norms when it comes to the distribution of metadiscourse features, with an increase in transition markers such as "accordingly" and "so" in translations, and a tendency to omit boosters, which are words or phrases that strengthen the author’s argument, and add hedges in English translations. In the genre of fiction, hedges and boosters can also be used to signal the writers and translator’s style [46]. Overall, these studies suggest that the representation of metadiscourse can vary significantly depending on the genre and language.

## 3. Research purpose and questions

There are several gaps in the current research on the use of metadiscourse features in translated languages. For example, there is a lack of studies on translation from Chinese and little research on the use of metadiscourse features across different genres. Additionally, previous studies have often only focused on one type of metadiscourse marker [17,38,39], making it difficult to get a full understanding of the differences between translated and non-translated texts. Moreover, it should be noted that a considerable number of these studies did not utilize any form of statistical analysis, making it exceedingly difficult to establish the significance of any observed differences [16,19]. To address these limitations, the current study uses two multi-genre balanced corpora—an original English corpus (FLOB) and the English sub-corpus of a parallel Chinese-English corpus (COCE)—to examine metadiscourse features. Details of the two corpora are given in the below section. The interactional metadiscourse analysis model developed by Hyland [47], which includes hedges, boosters, attitude markers, self-mentions, reader pronouns, and directives, is adapted to provide a comprehensive characterization of these features.

This study aims to investigate the following two research questions:

1. To what extent do the interactional metadiscourse features in translated English from Chinese differ from those in non-translated English texts?
2. Are there significant differences between translated and non-translated texts in terms of cross-genre variation of interactional metadiscourse features?

By addressing these research questions, this study aims to contribute to a better understanding of the representation of metadiscourse features in translated languages and the ways in which they may vary across genres and languages.

## 4. Methodology

### 4.1 Interactional metadiscourse framework

To examine how metadiscourse features are presented in translated texts compared to non-translated texts, the current study is largely based on the interactional metadiscourse analysis model developed by Hyland [47]. The proposed model comprises two dimensions, namely stance and engagement, which serve to analyse writers’ expressions of judgments, opinions, and commitments as well as their efforts to establish a connection with readers and draw attention to potential uncertainties. Specifically, the model includes four stance features and five engagement features. To align the model with our study’s objectives, we have included only two engagement markers, namely reader pronouns and directives, which are classified as lexical markers similar to other stance features proposed by Hyland [32]. These markers enable writers to express their perspectives explicitly and can be readily identified through fine-grained typologies such as corpus technology. In contrast, the other three engagement features in the framework, namely questions, appeals to shared knowledge, and personal asides, are context-dependent and less apparent than lexical items, and identifying them often necessitates the researcher’s evaluation rather than corpus techniques. Thus, we have adapted Hyland’s [47] model by excluding these three non-lexical features from the resources of engagement, as shown in Table 1. While Hyland [32] proposed a comprehensive framework for the classification of metadiscourse markers from both interactive and interactional perspectives, our study emphasized the delivery of interpersonal metafunction in translated language. Therefore, we have modified Hyland’s [47] model, which has a more specific categorization of stance and engagement markers, to examine how interactional metadiscourse features are employed in translated texts and how they differ from those in non-translated texts.

**Table 1:** Interactional metadiscourse model used in this study (adapted from Hyland [4,7]).

Interactional metadiscourse		Function	Example
Stance	Hedges	withhold commitment and open dialogue	might; perhaps; possible
	Boosters	emphasize certainty or close dialogue	in fact; definitely; clearly; demonstrate
	Attitude markers	express writer’s attitude to proposition	unfortunately; surprisingly; agree, prefer, appropriate, remarkable
	Self-mentions	explicit reference to author(s)	I; we; my; me; our
Engagement	Reader pronoun	explicitly build relationship with reader	you, your, inclusive we
	Directives	instruct the reader to perform an action or to see things in a way determined by the writer	Imperatives: see, note and consider Obligation modals: should, must, ought

### 4.2 Corpora

In this study, we compared the use and distribution of metadiscourse features in two comparable corpora: the Corpus of Chinese-English (COCE), which contains translated English texts, and the Freiburg-LOB Corpus of British English (FLOB), which contains non-translated English texts. By examining these two corpora, we aimed to identify similarities and differences in the use and distribution of interactional metadiscourse features in translated and non-translated English.

COCE is a multi-genre parallel Chinese-English balanced corpus that is comparable to FLOB in size and genres. It contains a subcorpus of source texts in Chinese and a subcorpus of target texts in English, and this study is based on the English subcorpus of COCE. The use of a parallel corpus such as COCE has several advantages over using a monolingual translated corpus. For example, it allows us to ensure that the translated subcorpus contains genuine translations rather than abridged or pseudo-translations. Moreover, because the English texts in COCE are translated from Chinese rather than multiple languages, our findings can be more specifically related to the Chinese-English language pair and context [48]. Like FLOB, COCE consists of 500 texts in four major genres, and the word token count does not include punctuation marks to ensure consistency with FLOB. The corpus design and statistics of the two corpora are shown in Table 2. For more information on COCE and FLOB, readers can refer to Liu and Afzaal [48]. To date, there has been no research that has investigated the use of metadiscourse features through the utilization of a multi-genre comparable corpus design. Therefore, it is essential to examine the degree to which translations differ from or resemble non-translations in their application of metadiscourse.

**Table 2:** Corpus design and statistics: FLOB and COCE.

Corpus	Genre	Number of texts	Average words per text	Total words
FLOB	News	88	2053.5	180,710
	General prose	206	2040.2	420,285
	Academic prose	80	2044.0	163,522
	Fiction	126	2069.1	260,706
Total		500	2051.7	1,025,223
COCE	News	88	2040.3	179,550
	General prose	206	2040.8	420,413
	Academic prose	80	2012.2	160,978
	Fiction	126	2019.8	254,495
Total		500	2028.3	1,015,436

In order to maximize comparability between the two corpora, the Corpus of Contemporary English (COCE) was carefully matched with the Freiburg-LOB Corpus (FLOB) in terms of size and composition. COCE consists of news (17.6%), general prose (41.2%), academic prose (16.0%), and fiction (25.2%), following the design of FLOB. The texts included in COCE were published during the same period as those in FLOB, predominantly in written translational English, and were collected from official and published sources that primarily targeted an English-speaking readership. Accordingly, following Baker’s corpus-based comparable research framework [3], we posit that the application of stance and engagement lexical markers can facilitate the comparison of writer-reader interactions between translated and non-translated languages.

### 4.3 Instrument

As computerized technology has advanced, automated text processing has become more widely used in linguistics, including tools such as the L2 Syntactic Complexity Analyzer [49,50], which allows for the automated extraction of syntactic complexity indicators, and the Multidimensional Analysis Tagger (MAT) [51], which replicates the Variation across Speech and Writing tagger of Biber [52] for the multidimensional functional analysis of English texts. In this study, we used the interactional metadiscoursal framework developed by Hyland [47] to calculate the types and frequencies of hedges, boosters, attitude markers, self-mentions, reader pronouns, and directives used in the Corpus of Chinese-English (COCE) and the Freiburg-LOB Corpus of British English (FLOB). However, due to the large size of these corpora, it would be impractical to rely solely on manual analysis. To address this issue, we adopted the Authorial Voice Analyzer (AVA) developed by Yoon [53], which has been confirmed to have a very high accuracy rate for quantifying interactional metadiscourse markers. According to Yoon, AVA is capable of capturing a large number of interactional metadiscourse markers and calculating normalized frequency values for these markers with high accuracy. It can also generate both token (total number of items) and type (number of unique items) statistics for metadiscourse features including boosters, hedges, and attitude markers. Table 3 outlines the functions of the AVA tool. For a more detailed description of AVA, readers can refer to Yoon [54].

**Table 3:** Interactional metadiscourse features from AVA [5,4].

Features	Token	Type	Normalized frequency
Hedge	✓	✓	Occurrences per 1000 words
Booster	✓	✓	
Attitude marker	✓	✓	
Self-mention	✓		
Reader pronoun	✓		
Directives	✓		

### 4.4 Analysis

In this study, we first used the Authorial Voice Analyzer (AVA) to extract frequency scores for six categories of stance and engagement features across the Corpus of Chinese-English (COCE) and the Freiburg-LOB Corpus of British English (FLOB) based on normalized frequency data. The raw statistics of these six categories are presented in Table 4. To examine whether there were significant differences in interactional metadiscourse features (six features) by translation status (translation and non-translation) and genres (press, prose, academic writing, and fiction), we conducted ANOVAs (Analysis of Variance). We first tested the normality of the six metadiscourse features in FLOB and COCE and found that only the hedge category was normally distributed. Therefore, in our analysis, we retained the original data for the hedge category, but logarithmically transformed the other five categories (booster, attitude, self-mention, reader pronoun, and directive) to address their positively skewed distributions. Since some features have a value of 0, we followed Field’s approach of adding a constant of 1 before logarithmic transformation. The mean, standard deviation, and skewness of the data after transformation are provided in Tables 4 and 5.

**Table 4:** Mean and standard deviation (in parentheses) for interactional discourse by genres.

Corpus	Genre	Hedge	Booster	Attitude marker	Self-mention	Reader	Directive
FLOB	News	13.17(4.59)	14.28(4.94)	12.82(5.61)	5.55(6.30)	5.85(5.49)	2.54(1.94)
	General Prose	14.26(5.87)	16.00(5.90)	11.92(4.67)	4.43(7.35)	7.52(10.15)	2.88(3.86)
	Academic prose	17.70(7.24)	17.04(9.03)	10.93(4.79)	1.70(2.87)	4.18(5.52)	3.07(2.61)
	Fiction	18.07(5.90)	18.39(6.04)	11.28(4.65)	25.79(18.83)	17.79(11.39)	2.65(1.61)
COCE	News	11.79(3.70)	12.54(3.38)	9.85(3.13)	4.01(3.62)	9.06(6.45)	1.94(1.17)
	General Prose	11.18(5.92)	14.62(6.43)	10.39(4.81)	15.12(18.30)	11.98(13.15)	3.17(3.23)
	Academic prose	10.16(5.44)	12.47(7.30)	9.50(3.66)	2.27(4.65)	3.28(4.27)	2.67(2.75)
	Fiction	15.27(5.22)	17.71(6.36)	12.69(6.08)	19.33(18.64)	15.44(11.73)	2.19(1.65)

**Table 5:** Mean and standard deviation values of interactional discourse by text categories after log transformation.

	hedge	Log10_boos-ter	Log10_atti-tude	Log10_self-mention	Log10_rea-der	Log10_direc-tive
N	1000	1000	1000	1000	1000	1000
Mean	13.87	1.19	1.05	0.74	0.83	0.49
Std. Deviation	6.18	0.18	0.18	0.55	0.46	0.25
Skewness	0.65	-0.73	-0.77	0.26	-0.23	0.26
Kurtosis	0.55	1.96	2.27	-1.1	-0.81	0.39

## 5. Results

A factorial between-groups Analysis of Variance (ANOVA) was conducted to investigate the effects of corpus and genre on the use of interactional metadiscourse features. Table 6 illustrates the interaction effects of these two independent variables on metadiscourse features. As shown in Table 6, significant interaction effects between corpus and genre were observed in all six metadiscourse categories, indicating that the effects of genre on the use of interactional metadiscourse markers may differ across the Corpus of Chinese-English (COCE) and the Freiburg-LOB Corpus of British English (FLOB). Although the interaction effects of corpus and genre were small in size, with partial eta squared ranging from .011 to .053, they demonstrated that genre has a unique effect on the use of interactional metadiscourse markers in the two corpora.

**Table 6:** Interaction effects between corpus and genre.

Feature	Corpus x Genre		
	*F*	*p*	*ηp* ^*2*^
Hedge	9.42	< .001*	.028
Booster	3.88	0.009*	.012
Attitude	7.10	< .001*	.021
Self-mention	18.52	< .001*	.053
Reader pronoun	6.17	< .001*	.018
Directive	3.74	0.011*	.011

* The *df* for the error term of all features was 992.

The main effects of each independent variable on the use of interactional metadiscourse features are shown in Table 7. Statistically significant effects of both corpus and genre were observed in almost all interactional metadiscourse features. In separate analyses for the effect of corpus, there was a significant difference between the two corpora in the use of hedges, *F* (1, 992) = 94.15, *p* < .001, *ηp*^*2*^ = .087; boosters, *F* (1, 992) = 29.14, *p* < .001, *ηp*^*2*^ = .029; attitude markers, *F*(1, 992) = 11.14, *p* = .001, *ηp*^*2*^ = .011; reader pronouns, *F*(1, 992) = 2.10, *p* = .023, *ηp*^*2*^ = .005; and directives, *F*(1, 992) = 4.37, *p* = .037, *ηp*^*2*^ = .004, with the largest effect size for hedges. Significant effects of genre were also found in various interactional metadiscourse features (hedges: *F* (3, 992) = 30.01, *p* < .001, *ηp*^*2*^ = .083; boosters: *F* (3, 992) = 20.43, *p* < .001, *ηp*^*2*^ = .058; attitude markers: *F* (3, 992) = 3.95, *p* = .008, *ηp*^*2*^ = .012; self-mentions: *F* (3, 992) = 89.80, *p* < .001, *ηp*^*2*^ = .307; reader pronouns: *F* (3, 992) = 87.99, *p* < .001, *ηp*^*2*^ = .210), with the largest effect size for self-mentions. Our analysis showed that both corpus and genre had a significant impact on the use of interactional metadiscourse features in the two corpora.

**Table 7:** Main effects of corpus and genre.

Feature	Corpus			Genre		
	*F*	*p*	*ηp* ^*2*^	*F*	*p*	*ηp* ^*2*^
Hedge	94.15	< .001*	.087	30.01	< .001*	.083
Booster	29.14	< .001*	.029	20.43	< .001*	.058
Attitude	11.14	.001*	.011	3.95	.008*	.012
Self-mention	2.10	.147	.002	89.80	< .001*	.307
Reader pronoun	5.17	.023*	.005	87.99	< .001*	.210
Directive	4.37	.037*	.004	1.55	.201	.005

*p values are significant with the Bonferroni correction (*p* < .05).

**Fig 1:**
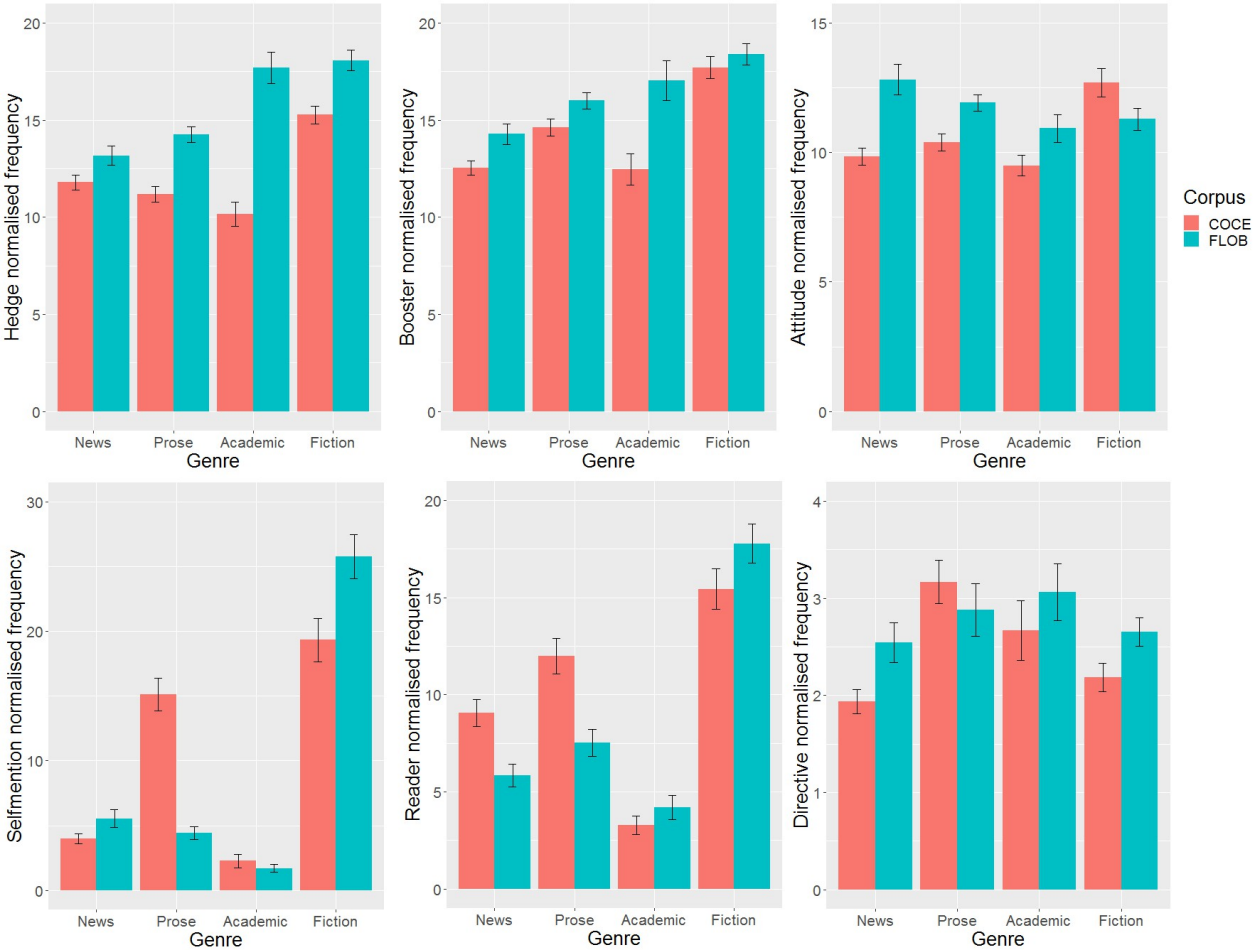
Normalized frequency comparison of interactional markers in COCE and FLOB.

**Fig 2:**
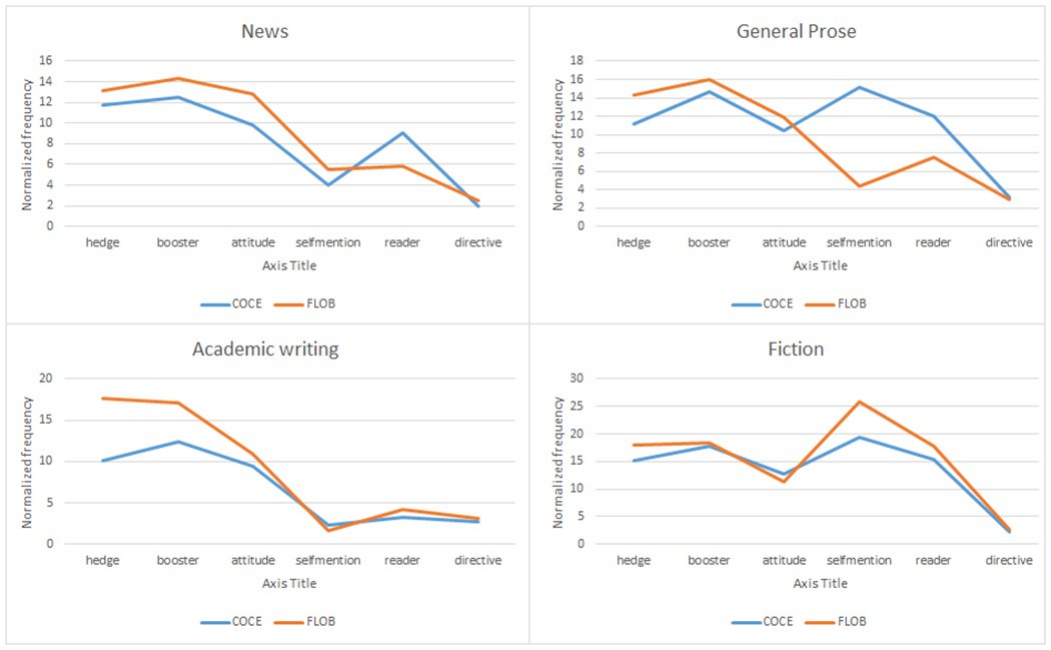
Variation of interactional markers between COCE and FLOB.

Simple effects analyses were conducted to further examine the interaction between corpus and genre. The results showed that there was significantly more use of hedges in FLOB than in COCE in general prose with a medium effect size (*p* < .001, *d* = .522), and a similar trend was also found in academic prose with a large effect size (*p* < .001, *d* = 1.177), and in fiction with a medium effect size (*p* < .001, *d* = .503). In terms of boosters, FLOB recorded significantly more use of boosters than COCE in general prose with a small effect size (*p* = .001, *d* = .224), and in academic prose with a medium effect size (*p* < .001, *d* = .557). As for attitude markers, significantly more attitude markers were found in FLOB than in COCE in two genres: news with a medium effect size (*p* = .001, *d* = .654) and general prose with a small effect size (*p* < .001, *d* = .323). In addition, the use of self-mentions in general prose in FLOB was significantly lower than in COCE with a medium effect size (*p* < .001, *d* = .767), but significantly higher in fiction in FLOB than in COCE with a small effect size (*p* = .005, *d* = .345). Regarding the use of reader pronouns across various genres, it was observed that the frequency of reader pronouns in FLOB was significantly lower than that in COCE, specifically in news articles with a medium effect size (*p* = .005, *d* = .536) and in general prose with a small effect size (*p* < .001, *d* = .380). A significant difference was only observed between FLOB and COCE in directives in the genre of fiction. Figs 1 and 2 show the by-feature and by-genre comparisons of the use of metadiscourse features between COCE and FLOB. A summary of the significant differences in the use of interactional markers based on normalized frequency across genres between FLOB and COCE is presented in Table 8.

**Table 8:** Significant differences in the use of interactional metadiscourse features across genres between FLOB and COCE.

	Hedge	Booster	Attitude marker	Self-mention	Reader pronoun	Directive
News			>		<	
General prose	>	>	>	<	<	
Academic prose	>	>				
Fiction	>			>		>

* The inequality signs in the statistical analysis indicate significant differences with the Bonferroni correction (p < .05/2 = .025). The ">" symbol signifies that FLOB values are higher than COCE values, while the "<" symbol denotes that FLOB values are lower than COCE values.

To further examine the use of metadiscourse features between genres in COCE and FLOB, post-hoc comparisons with Bonferroni correction were conducted, with the alpha level adjusted to .05/6 = .0083. The results of these comparisons are shown in Table 9. The analysis revealed that translated news differed significantly from translated general prose in the use of self-mentions and directives, and that this difference was also observed in self-mentions in the non-translated pair. Additionally, news differed significantly from academic writing in terms of self-mentions and reader pronouns in both the translated and non-translated texts. In terms of cross-genre variation, translated news also differed significantly from fiction in terms of attitude markers, while non-translated news differed significantly from fiction in hedges, boosters, self-mentions, and reader pronouns.

**Table 9:** Bonferroni post-hoc comparisons of interactional metadiscourse features across genres in COCE/FLOB.

Genres		Hedge	Booster	Attitude marker	Self-mention	Reader	Directive
News		-/-	-/-	-/-	*/*	-/-	*/-
	Academic prose	-/*	-/-	-/-	*/*	*/*	-/-
	Fiction	*/*	*/*	*/-	*/*	*/*	-/-
General Prose	Academic prose	-/*	*/-	-/-	*/*	*/*	-/-
	Fiction	*/*	*/*	*/-	*/*	*/*	*/-
Academic prose	Fiction	*/-	*/-	*/-	*/*	*/*	-/-

*Note*: The alpha level for the Bonferroni correction has been adjusted to .05/6 = .0083. In the table, * represents a significant between-genre difference (*p* < .0083); - represents no significant between-genre difference (*p* ≥ .0083). In each cell, the symbols to the left and right of / represent the level of significance for COCE and FLOB, respectively.

A varied picture emerged when examining the differences between general prose and other genres in COCE and FLOB. General prose differed significantly from academic writing in terms of self-mentions and reader pronouns. Translated general prose differed significantly from translated academic writing in terms of boosters, but not in other metadiscourse features. In contrast, non-translated general prose differed significantly from non-translated academic writing in terms of hedges, but not in other metadiscourse features. In terms of cross-genre variation between translated general prose and fiction, significant differences were observed in all metadiscourse features. When comparing attitude markers between genres in COCE, significant differences were observed, while no significant differences were found in FLOB. Finally, significant differences between academic writing and fiction were observed in COCE in almost all metadiscourse features, including hedges, boosters, attitudes, self-mentions, and reader pronouns. In FLOB, significant differences were only observed in self-mentions and reader pronouns.

## 6. Discussion

The present study employed a corpus-based approach to compare the use of interactional metadiscourse features in translated and non-translated English texts. The corpus of Chinese-English (COCE) and the Freiburg-LOB Corpus of British English (FLOB) were analyzed using the Authorial Voice Analyzer (AVA) tool [53] to examine six metadiscourse features. The results showed that there were statistically significant differences in the use of interactional metadiscourse features between the two corpora and across genres. These findings suggest that while translated English exhibits some similarities in terms of the use of metadiscourse features, it also displays unique characteristics in the achievement of the interpersonal metafunction across different genres.

### 6.1 RQ1: To what extent do the interactional metadiscourse features in translated English from Chinese differ from those in non-translated English texts?

Our study aimed to answer the question of whether interactional metadiscourse features differ between translated English from Chinese and non-translated English texts. The results revealed significant differences in the frequencies of five metadiscourse features, namely hedge, booster, attitude, reader pronoun, and directive, except for self-mention. These differences were observed regardless of genre. Notably, translated English texts exhibited a tendency to underrepresent three out of four stance markers (i.e., hedge, booster, and attitude), in terms of both tokens and types. According to Biber and colleagues [55], stance involves expressing "personal feelings, attitudes, value judgments, or assessments" in addition to the propositional content of a text. The underuse of stance features in translated English texts from Chinese, as indicated by the quantification of both types and tokens, signifies a potential dearth of the attitudinal dimension. This dimension comprises interactional features that facilitate writers in projecting their self-image and conveying their evaluations, viewpoints, and commitments. This lack of attitudinal dimension may impact the way these texts are perceived and understood by readers. In contrast, translated texts tend to be overrepresented with reader pronouns, but underrepresented in terms of directives. Hyland notes that both stance and engagement contribute to the interpersonal dimension of discourse [47], and the underrepresentation of these interactional metadiscourse features implies that, to some extent, translation as a communicative activity mediated by language lacks the "community-sensitive linguistic resources" that writers employ to express themselves, their viewpoints, and their readers [47].

### 6.2 RQ2: Are there significant differences between translated and non-translated texts in terms of cross-genre variation of interactional metadiscourse features?

In our second research question, we aimed to investigate the differences in cross-genre variation of interactional metadiscourse features between translated and non-translated English. Our findings indicated that the translation status effect, which is the difference in metadiscourse features between translated and non-translated texts, varied depending on the genre. We observed an interaction effect of corpus and genre, suggesting that the translation status effect differs across genres for all six metadiscourse features examined. Pairwise within-genre comparisons revealed a mixed picture for both stance and engagement features, providing a more nuanced understanding of how metadiscourse features are used in different genres. In the news genre, prior research has found that translations tend to employ fewer attitude markers [37,39]. Attitude markers are linguistic devices utilized to convey the emotions and opinions of the writer, such as surprise and frustration [47]. They can take various forms, including verbs like "agree" and "prefer", adverbs such as "dramatically" and "rightly", and adjectives like "dramatic" and "bizarre". These devices are often employed to influence readers to adopt the writer’s standpoint. Table 10 provides a list of the top 20 overused attitude markers in the news category. The analysis shows that a majority of the overused items in native news articles are adjectives.

**Table 10:** The top 20 overused attitude markers in news category of FLOB compared with that of COCE.

Word*	FLOB	COCE	Log Likelihood	ELL^#^
worst	22	2	19.37	< 0.001
dramatic*	28	5	17.53	< 0.001
appalling*	11	0	15.18	< 0.001
sensibl*	10	0	13.80	< 0.001
dreadful*	9	0	12.42	< 0.001
decent	15	2	11.17	< 0.001
convincing*	8	0	11.04	< 0.001
brilliant*	24	7	9.75	< 0.001
modest*	11	1	9.69	< 0.001
rightly	16	3	9.68	< 0.001
bizarre*	7	0	9.66	< 0.001
justified*	7	0	9.66	< 0.001
essential*	25	8	9.08	< 0.001
excellent*	26	9	8.51	< 0.001
embarrassing*	10	1	8.49	< 0.001
fairly	10	1	8.49	< 0.001
disgraceful*	6	0	8.28	< 0.001
marvellous*	6	0	8.28	< 0.001
pleasant*	6	0	8.28	< 0.001
respectabl*	6	0	8.28	< 0.001

*Note*:

* The AVA software utilizes regular expressions to extract words and their corresponding variants.

^#^ The Effect Size for Log Likelihood (ELL) is calculated according to Johnston et al. [56] and ranges from 0 to 1.

This measure is interpreted as the proportion of the maximum deviation between observed and expected proportions, which is straightforward.

The examination of interactional metadiscourse features in translated English texts, as presented in Table 8, reveals certain general tendencies in their usage. Notably, the influence of genre on the representation of metadiscourse features in translated English is evident. Metadiscourse features in translated literary texts, including general prose and fiction, exhibit greater divergence than those in non-literary texts such as news and academic prose. This finding aligns with similar observations reported in previous studies [57,58]. Bielsa [59] contends that for non-literary text types, such as news reports, which are primarily informative, minimal register and stance shifts are necessary during the translation process. This assertion may account for the lower frequency of metadiscourse variances observed in translated news. However, our analysis of academic prose indicated that native English displays more hedges and boosters than translated English (see Table 11), indicating variations in the presentation of academic discourse between English and Chinese [13,14]. Therefore, the genre of the source text plays a significant role in shaping the use and distribution of metadiscourse features in translated English texts. The results described above suggest that the role of metadiscourse in translated texts may be influenced by a variety of factors, including genre-specific norms and conventions [39], as well as the challenges and constraints faced by translators [21]. Genre-specific norms and conventions refer to the expectations and conventions that are specific to a particular genre or type of text. For example, academic prose tends to have different norms and conventions for the use of metadiscourse than news articles or fiction. These norms and conventions may be shaped by the intended audience, purpose, and context of the text, and may influence how metadiscourse is used in translated texts [44]. Translation is a complex process that involves the transference of meaning from one language to another, and is subject to various factors such as the source text, the target language and audience, as well as the translator’s individual competencies and expertise. Consequently, translators may encounter diverse challenges and constraints that can influence their utilization of metadiscourse in the translated text. For example, they may need to consider how to convey the same level of politeness, formality, or tone as in the source text, or how to adapt the text to fit the conventions and expectations of the target audience. These challenges and constraints can influence the way metadiscourse is used in translated texts, and may contribute to the differences observed between translated and non-translated texts.

**Table 11:** The top 20 overused hedges and boosters in academic prose of FLOB compared with that of COCE.

**Hedges** *	**FLOB**	**COCE**	**Log Likelihood**	**ELL** ^#^
may	275	102	79.75	< 0.001
(?<!im)(possible|possibly|possibility|possibilities)*	126	45	38.69	< 0.001
assumption*	35	3	31.19	< 0.001
suggest(ed|s|ing)*	96	35	28.58	< 0.001
(?<!im)(probable|probably|probability|probabilities) *	61	19	22.54	< 0.001
suppos(e|ing)*	35	7	19.94	< 0.001
rather(?! than)*	91	41	18.64	< 0.001
perhaps	42	12	17.18	< 0.001
likely	66	27	16.27	< 0.001
unlikely	15	1	14.48	< 0.001
(?<!un)seem(?!ly) *	26	6	13.16	< 0.001
(i|we)\s\w*\s?\w*\s?argue*	9	0	12.34	< 0.001
(i|we)\s\w*\s?\w*\s?assume*	13	1	12.02	< 0.001
postulat(e|ing)*	8	0	10.97	< 0.001
seen as*	17	3	10.60	< 0.001
might	69	36	10.04	< 0.001
in principle*	11	1	9.60	< 0.001
(?<!in|un)(arguable|arguably)*	7	0	9.59	< 0.001
(presumable|presumably)*	7	0	9.59	< 0.001
often	91	53	9.56	< 0.001
**Boosters** *	**FLOB**	**COCE**	**Log Likelihood**	**ELL** ^#^
evident*	88	19	47.17	< 0.001
(?<!not) much*	118	50	27.27	< 0.001
(maximal|maximum)*	26	2	24.03	< 0.001
indeed	58	19	20.09	< 0.001
at all*	44	16	13.15	< 0.001
(?<!un)(true|truly|truth)*	68	32	12.70	< 0.001
the fact that*	41	16	10.96	< 0.001
clearly	51	23	10.43	< 0.001
must	130	84	9.26	< 0.001
more so*	42	19	8.53	< 0.001
very	199	143	8.35	< 0.001
badly	6	0	8.22	< 0.001
obvious*	38	17	7.90	< 0.001
(?<!im)perfect*	16	4	7.52	< 0.001
particularly	61	34	7.36	< 0.001
highly	34	15	7.27	< 0.001
(certain that|certainly)*	26	10	7.12	< 0.001
(absolute|absolutely)*	11	2	6.72	< 0.001
(?<!im)precise*	11	3	4.74	< 0.001
readily	16	6	4.56	< 0.001

*Note*:

* The AVA software utilizes regular expressions to extract words and their corresponding variants.

^#^ The Effect Size for Log Likelihood (ELL) is calculated according to Johnston et al. [56] and ranges from 0 to 1.

This measure is interpreted as the proportion of the maximum deviation between observed and expected proportions, which is straightforward.

## 7. Conclusion and limitations

### 7.1 Implications for translation research and teaching

This study investigated the interactional metadiscourse features present in translated English, and compared them to those found in originally produced English using two genre-balanced corpora. Overall, the results indicated that English translations had a significantly lower frequency and variety of metadiscourse features when compared to non-translated native writing. This finding is consistent with the majority of studies that have explored the metadiscourse features of translated languages. Based on the distribution of metadiscourse features in both translated English and native English writing, our study demonstrated that translation is categorically different from non-translation in terms of the interpersonal dimension shaped by interactional metadiscourse features. Specifically, our analysis revealed an under-representation of two important stance markers, namely hedges and boosters, in translated academic English when compared to original English in the same genre. This outcome aligns with earlier studies on translated and non-translated academic writing from diverse disciplines, which indicates a greater prevalence of hedges in the latter [60,61]. In light of our findings, it is crucial to give equal consideration to the translation of interpersonal values alongside propositional content. As noted by Hyland [11], Anglo-American academic writers use hedges to demonstrate prudence, tentativeness, and commitment when presenting propositions and arguments to disciplinary communities. However, translated academic writing may differ in the "truth" value of their findings due to different evidentiary and epistemic traditions. Therefore, it is essential for writers and translators to incorporate stance markers to ensure that their claims display a plausible relationship with reality using the epistemic conventions and argument forms of their disciplines. Incorporating stance markers in translation training can help students develop a better understanding of the importance of interpersonal values in academic writing and the strategies for expressing them. Such training is particularly important in helping students develop the necessary skills to engage in debate and argumentation as a process of constructing knowledge [62].

The implications of the current study for metadiscourse features in translated languages are multifaceted. To start with, the results of this study provide a more thorough understanding of these features through the lens of the representation of the interpersonal metafunction. Our findings align with single-genre studies such as those by Peterlin [17] and Skrandies [45], which have shown that metadiscourse features are represented differently in translation and non-translation. Our study adds to this body of research by demonstrating that translated texts tend to have similar tendencies, such as under-use of certain classes of words, as previously identified by some studies [63,64]. However, the over-representation of self-mentions and reader pronouns in translated English news and general prose is a new finding that may warrant further investigation. This finding suggests that there may be unique patterns of engagement between metadiscourse features and genres in translated texts, and could potentially open up new avenues for exploration in this area.

Secondly, the under-representation of metadiscourse features in translated English from Chinese may be due to the translators adhering too closely to the source text conventions and norms. As Hinds [65] notes, in English, the writer plays a dominant role in effective communication, while in Asian languages such as Chinese, Japanese, and Korean, the reader is also deeply involved in the communication process. In Asian languages, writers tend to be more implicit and allow readers to interpret language hints and nuances, rather than spelling things out explicitly. Translated English has been described as a unique type of language production that shares some similarities with L2 varieties of English [66]. Research has shown that effective metadiscourse use is typically associated with skilled writers and speakers who are able to create a mutual frame of reference and anticipate how their purposes will be received by their audience [14,32]. The current study demonstrates that advanced writers represented in the FLOB corpus (a corpus of written British English) used a greater number and variety of metadiscourse features than translators in the COCE corpus (a corpus of written English by Chinese speakers). This suggests that translated English texts may result in an under-representation of metadiscourse features compared to non-translated English texts, and may share some similarities with L2 writing by novice writers [66–68], which is characterized by a misuse or underuse of effective metadiscourse features. When considering the metadiscourse functions of translated texts more broadly, it is important to recognize the influence of culture on language. As Hyland [11] notes, people from different cultures may have different expectations about the logical organization of written texts, and what is seen as logical, engaging, relevant, or well-organized in writing may vary across cultures. The findings of the current study suggest that translation, as a norm-governing activity, is influenced by the social and cultural practices and traditions represented in both the source and target languages. This underscores the complexity and uniqueness of the translation.

Finally, genre may play a role in the exploration of metadiscourse features in translated languages [69]. The results of this study show that interpersonal metadiscourse features have cross-genre representations. While we observe an under-representation of stance features such as hedges and boosters in translated academic prose and newspapers, similar to the findings of many previous studies, we also find an overuse of some engagement features in translated newspapers and general prose. This suggests that investigations into metadiscourse features in translated languages should not ignore the influence of register or genre if we aim to fully understand the nature of this translation phenomenon. Previous studies have often been limited to a single genre, but the current study, which is based on a comparison of two balanced corpora (each composed of four genres), provides more robust evidence. The use of a multi-genre corpus has enabled us to obtain a more holistic view of metadiscourse features in translated texts. In the field of discourse analysis, previous studies of metadiscourse features in translation have often focused on isolated features. While these studies have provided valuable insights into the use of metadiscourse features in translation and non-translation, they have not been able to give a complete picture of how translation is unique in terms of metadiscourse features. The current study takes a more comprehensive approach, examining multiple metadiscourse features across a range of genres, and as such, provides a more nuanced understanding of the role of metadiscourse in translated texts.

### 7.2 Limitations

Our findings suggest that metadiscourse measures are useful for understanding the interpersonal metafunction in translated language, or "the third code," across different genres. However, it is important to note that the present study’s results are limited to English translations from Chinese, primarily translated by Chinese speakers. Therefore, our corpus may not be representative of translations produced by translators with different language backgrounds. Other variables, such as the direction of translation and translators’ language competence, may also influence the characteristics of translated texts. Future studies could explore other metadiscourse features, such as questions, appeals to shared knowledge, and personal asides, in translated language across different language pairs and genres to provide a more comprehensive understanding of the characteristics of translated languages in terms of metadiscourse use.
